# Left Ventricular Myxoma in a Young Adult: An Unusual Presentation of a Rare Cardiac Tumor

**DOI:** 10.7759/cureus.110936

**Published:** 2026-06-15

**Authors:** Juan Pablo Montoya L, María Camila Vélez, Juan Camilo Quintero, Alejandro Villamizar, Valentina Palacio-Arango

**Affiliations:** 1 Cardiology, Clínica Especializada EMMSA, Bello, COL; 2 General Medicine, Clínica Especializada EMMSA, Bello, COL; 3 Epidemiology, Clínica Especializada EMMSA, Bello, COL

**Keywords:** atypical presentation, cardiac surgery procedures, intracardiac myxoma, left ventricular mass, transthoracic echocardiography

## Abstract

Cardiac myxomas are rare primary tumors, generally benign, and predominantly located in the atria, especially the left. Their clinical presentation is variable, and they can manifest with embolic events, intracardiac obstruction, or as an incidental finding. We present a case of a 25-year-old patient with fatigue and atypical chest pain, in whom transthoracic echocardiography revealed a pedunculated, irregular-bordered, highly mobile intracavitary mass in the left ventricle, attached to the interventricular septum. Given the unusual location and the potential risk of complications, complete surgical resection was performed, with no adverse postoperative events. Histopathological examination confirmed the diagnosis of cardiac myxoma. This case highlights an uncommon presentation of this entity, underscoring the importance of considering cardiac tumors in the differential diagnosis of ventricular masses and the fundamental role of echocardiography in their timely detection and therapeutic planning.

## Introduction

Primary cardiac tumors are rare, with an estimated prevalence of 0.01-0.03% in the general population, according to autopsy series [1,2]. Of these, approximately 90% are benign, with myxomas being the most common histological type, representing about 80% of cases [2]. Cardiac myxomas are neoplasms of heterogeneous morphology that may contain fibrous, cystic, and hemorrhagic components. They predominantly affect adults between the fifth and sixth decades of life, more frequently in women. Their most common location is the left atrium (around 80%), followed by the right atrium, while their presentation in the ventricles is infrequent [1,2].

The clinical presentation is variable and includes syncopal episodes, systolic murmurs secondary to left ventricular outflow tract (LVOT) obstruction, and embolic phenomena; however, a significant number of cases may be asymptomatic and diagnosed incidentally on imaging [3-5]. We present a case of a young patient with a myxoma in an unusual location in the left ventricle, diagnosed by echocardiography and confirmed by histopathological examination, which was successfully treated by surgical resection. This case highlights the importance of considering this entity in the differential diagnosis of intracardiac masses, especially in atypical locations.

## Case presentation

A 25-year-old man with a history of bilateral Leydig cell carcinoma treated by inguinal orchiectomy, followed by magnetic resonance imaging with no evidence of oncological progression, presented with a four-month history of sharp, stabbing chest pain in the left precordial region, unrelated to exercise and associated with fatigue. Upon admission and throughout hospitalization, the patient remained hemodynamically stable, with blood pressure within normal ranges, a normal heart rate, and adequate oxygen saturation on room air. The physical examination revealed no significant findings, including heart murmurs, adventitious sounds, or signs of fluid overload or heart failure. The electrocardiogram showed sinus rhythm with preserved atrioventricular conduction and no acute ischemic changes. Laboratory studies, including complete blood count, renal function tests, serum electrolytes, and biomarkers of myocardial injury, were within normal limits. No constitutional symptoms, embolic events, or associated infectious manifestations were documented.

**Figure 1:**
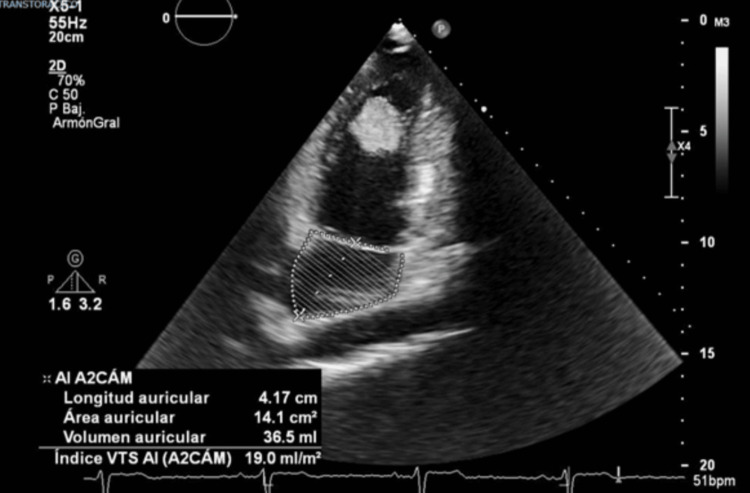
Apical two-chamber view showing a large, mobile, pedunculated 23 mm mass within the left ventricle, attached to the interventricular septum.

**Figure 2:**
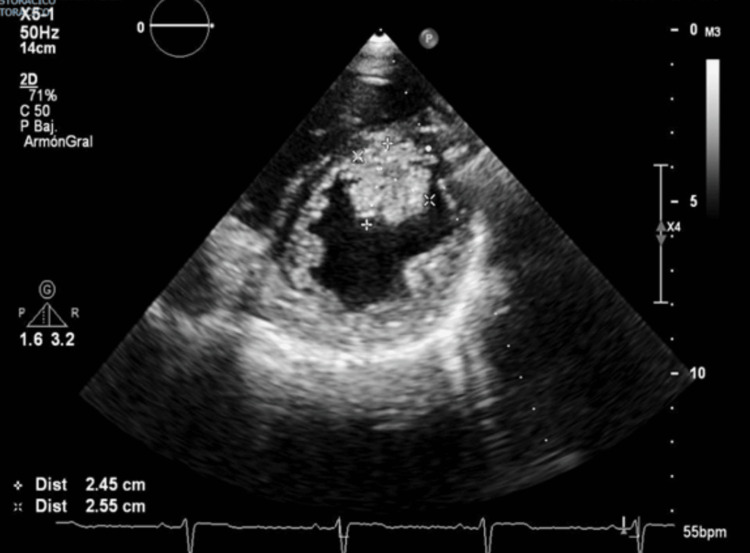
Parasternal short-axis view demonstrating a heterogeneous, intracavitary left ventricular mass with defined borders and measured dimensions of approximately 2.45 × 2.55 cm.

**Figure 3:**
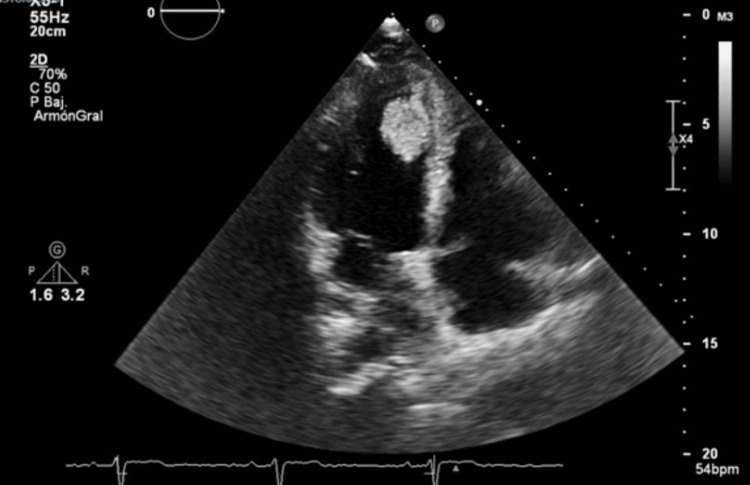
Apical four-chamber view showing a mobile intracavitary mass within the left ventricle.

**Figure 4:**
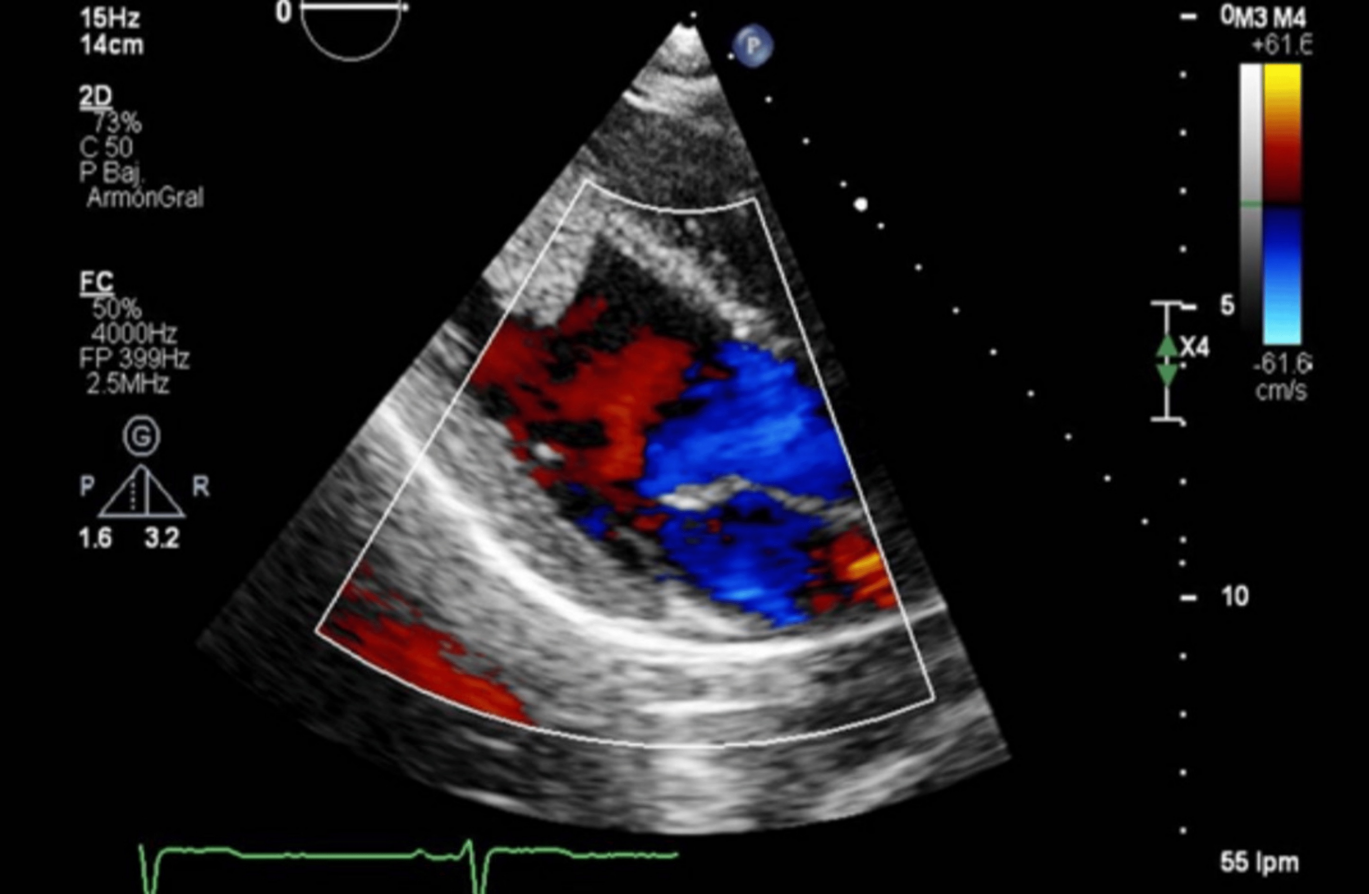
Color Doppler in long parasternal view showing preserved intracardiac flow without evidence of valvular dysfunction.

In this case, metastatic cardiac disease was considered due to the patient’s oncological history; however, the absence of disease progression on follow-up imaging studies, together with the echocardiographic appearance of a pedunculated and highly mobile intracavitary lesion, made a primary cardiac tumor more likely. As part of the initial workup, a transthoracic echocardiogram was performed, demonstrating a left ventricle of normal size and preserved global systolic function, with a left ventricular ejection fraction ranging between 55% and 60%, without segmental wall motion abnormalities or diastolic dysfunction. A pedunculated intracavitary mass measuring approximately 23 mm was identified within the left ventricle, attached to the anterior interventricular septum at the junction of its mid and distal thirds (Figure 1). The lesion was densely echogenic, highly mobile, and exhibited irregular borders, features suggestive of high embolic potential (Figures 2, 3). No evidence of left ventricular outflow tract obstruction or significant intracardiac gradient was observed. Right ventricular size and systolic function were preserved (tricuspid annular plane systolic excursion {TAPSE} 25 mm; S’ wave 14 cm/s). Cardiac chambers were otherwise normal in size, and no significant valvular abnormalities were identified, except for mild pulmonary regurgitation without indirect signs of pulmonary hypertension. Doppler evaluation demonstrated normal filling pressures and preserved intracardiac flow dynamics (Figure 4).

In the context of the presurgical evaluation, coronary angiography was performed, which revealed a small arterio-arterial fistula into the left ventricular cavity, without indication for intervention. Anticoagulation was initiated, and a follow-up echocardiogram was performed one week later, showing no changes in the size or characteristics of the lesion (Figure 2).

Given the persistence of the mass, the patient was evaluated by cardiovascular surgery and underwent surgical resection through a median sternotomy under cardiopulmonary bypass with aortic and right atrial cannulation. After aortic cross-clamping, myocardial protection was achieved with antegrade cardioplegia administered through the aortic root. Initial surgical exposure was performed through a left atriotomy; although the mass could be visualized, the exact site of implantation was not clearly identified. Therefore, an aortotomy was subsequently performed, allowing improved visualization of the lesion within the left ventricular cavity. A highly mobile mass measuring approximately 30 mm, with gross features suggestive of myxoma, was identified arising from a pedunculated implantation over a trabecular structure measuring approximately 3 mm. The tumor was carefully dissected and completely resected under direct visualization in order to minimize fragmentation and potential embolization. Inspection of the remaining endocardial surface did not reveal additional masses or abnormal lesions. The aortotomy and left atriotomy were closed in two layers, followed by de-airing maneuvers and successful weaning from cardiopulmonary bypass without difficulty. Intraoperative transesophageal echocardiography demonstrated preserved biventricular function, absence of significant valvular dysfunction, no intracardiac shunts, and no evidence of residual tumor. In the immediate postoperative period, the patient remained hemodynamically stable in sinus rhythm, without requiring vasoactive or mechanical circulatory support. Postoperative recovery was favorable, with preserved respiratory function, minimal mediastinal drainage, adequate urine output, and no evidence of early surgical complications.

Following surgical resection, the patient had an uneventful postoperative course and was discharged home nine days after surgery. He experienced complete resolution of his presenting symptoms and remained clinically stable during follow-up. Serial outpatient evaluations by cardiology and cardiovascular surgery were conducted over a nine-month period. Histopathological examination confirmed the diagnosis of cardiac myxoma. Immunohistochemical analysis demonstrated positivity for calretinin, smooth muscle actin (SMA), and CD31, with focal expression of S100 and negative staining for cytokeratin cocktail, findings supportive of the diagnosis of cardiac myxoma.

## Discussion

Myxomas are the most common benign primary tumors of the heart, although they remain rare [1]. Their predominant location is the left atrium; however, left ventricular involvement is exceptional, representing approximately 3-4% of reported cases [1,4]. This atypical location presents a diagnostic challenge, requiring consideration of a broad differential diagnosis that includes thrombi, vegetations, papillary fibroelastoma, primary cardiac sarcomas, and other intracardiac masses [1,2]. In this case, the differential diagnosis also included metastatic cardiac involvement due to the patient's oncological history. However, the absence of tumor progression on follow-up oncological imaging studies, along with echocardiographic findings of a pedunculated, highly mobile intracavitary mass adhered to the interventricular septum, favored the diagnosis of a primary cardiac tumor rather than metastatic disease. Furthermore, the lesion demonstrated preserved mobility without features suggestive of a mural thrombus, such as a broad-based adhesion or reduced movement [1,2]. Papillary fibroelastoma was considered less likely, as these tumors usually originate in valvular structures and are generally smaller [2,5]. Likewise, the absence of infiltrative features, myocardial invasion, or aggressive clinical behavior made primary cardiac sarcoma less likely [1,5]. Infectious vegetation was also considered unlikely, given the absence of fever, constitutional symptoms, valvular involvement, or inflammatory abnormalities on laboratory tests, and the negative blood cultures [2,5]. Cardiac magnetic resonance imaging was not performed because it was not routinely available at our institution during the patient's evaluation. However, transthoracic echocardiography, intraoperative transesophageal echocardiography, surgical findings, and definitive histopathological examination provided sufficient diagnostic characterization of the lesion. Before 1951, the diagnosis of intracardiac tumors could only be made by autopsy; the first premortem diagnosis of a left atrial myxoma was made by angiography in 1952, and the first diagnosis by echocardiography was made in 1959 [4].

The clinical manifestations of myxomas depend on their size, location, and mobility. It has been reported that approximately 10% of patients are asymptomatic and up to 30% present with nonspecific symptoms, while 60% develop obstructive, embolic, and/or constitutional manifestations [6,7]. In this context, clinical suspicion may be low, especially in young patients without cardiovascular risk factors, which may delay diagnosis [8].

Transthoracic echocardiography remains the initial diagnostic tool of choice, as it allows for the identification of morphological characteristics such as size, mobility, and implantation site [2,8]. Other complementary diagnostic techniques, such as computed tomography and cardiac magnetic resonance imaging, allow for better characterization of the mass, its extent, and its relationship to adjacent structures [6]. Additionally, transesophageal echocardiography offers higher spatial resolution, proving particularly useful in the evaluation of small or complexly located masses [9,10]. Multimodal imaging has gained importance in recent years, enabling improved surgical planning and tissue characterization of cardiac masses [11,12].

Minor differences in tumor dimensions between imaging studies and intraoperative assessment likely reflect the intrinsic limitations of echocardiographic measurements in highly mobile intracavitary masses, which may vary according to imaging plane and tumor orientation [11,12]. In our case, while transthoracic echocardiography estimated the lesion at approximately 23 mm, direct intraoperative visualization described the mass as measuring close to 30 mm. Likewise, the apparent discrepancy regarding the implantation site may be explained by the complementary nature of imaging and surgical findings. Echocardiography localized the lesion to the anterior interventricular septum at the junction of its middle and distal thirds, whereas intraoperative inspection identified a pedunculated attachment arising from a trabecular structure adjacent to the septal region, providing a more detailed anatomical characterization of the tumor base [11,12].

Surgical resection is the treatment of choice and should be performed early due to the risk of embolization and intracardiac obstruction. Tumors located in the left ventricle have a higher risk of morbidity and mortality from systemic embolization or obstruction of the left ventricular outflow tract and coronary circulation [1,9]. Several factors have been associated with an increased embolic risk in cardiac myxomas, including high tumor mobility, pedunculated implantation, irregular or friable surfaces, and ventricular location [11,12]. In the present case, the lesion exhibited several of these high-risk features, particularly marked mobility, irregular borders, and left ventricular involvement, supporting the indication for early surgical resection despite the patient’s relatively nonspecific symptoms.

Contemporary series have demonstrated that complete surgical resection is associated with excellent long-term results, with low operative mortality and a low recurrence rate in sporadic cases [12]. However, myxoma resection does not require complete removal of the ventricular wall, as recurrence is unlikely when limited resections are performed [1,2]. The prognosis for patients with cardiac myxoma is generally favorable, with a 20-year survival rate of 85% [9]. However, factors such as atypical location, tumor size, and the presence of embolic complications can influence the clinical outcome [9]. Although recurrence is uncommon after complete surgical resection, long-term follow-up with serial echocardiographic surveillance is recommended, particularly in patients with atypical tumor locations such as the left ventricle, due to the potential risk of recurrence and late postoperative complications [5,11].

In this context, the present case is noteworthy because it involves a myxoma located in the left ventricle of a young patient, which is an unusual presentation. This type of case highlights the importance of considering cardiac tumors in the differential diagnosis of intracavitary masses, even in patients with atypical symptoms, as well as the value of a comprehensive and multidisciplinary diagnostic approach to optimize management and reduce the risk of complications [11,12].

Given the exceptional rarity of left ventricular myxoma, the available evidence is largely limited to isolated case reports and small case series. To provide a broader clinical perspective, we summarized previously published cases in adult patients in Table 1. Pediatric cases (< 18 years) were excluded because the profile of primary cardiac tumors differs substantially with age. While myxoma is the most common primary cardiac tumor in adults, rhabdomyoma predominates in the pediatric population [13], whereas cardiac myxomas are uncommon and more frequently associated with familial or syndromic forms, particularly Carney complex [2,11]. Therefore, to maintain a clinically homogeneous study population and facilitate meaningful comparisons with the present case, the literature review was restricted to adult patients. This compilation highlights the extremely low prevalence of left ventricular myxomas and the heterogeneity of their clinical presentation, while also emphasizing the diagnostic and therapeutic challenges associated with this unusual location.

**Table 1:** Case reports of left ventricular myxoma in adults. TTE: transthoracic echocardiography; TEE: transesophageal echocardiography; LVOT: left ventricular outflow tract; LVEF: left ventricular ejection fraction; NYHA: New York Heart Association; CPB: cardiopulmonary bypass; LAD: left anterior descending artery; PET/CT: positron emission tomography/computed tomography; AV: atrioventricular; SMA: smooth muscle actin; 18F-FDG: fluorine-18 fluorodeoxyglucose

Studies	Sex, age (years)	Relevant history	Clinical presentation (symptoms and physical examination)	Tumor characteristics (location, attachment, and size)	Diagnosis and surgical approach	Histopathology	Outcome, recurrence, and follow-up
Kawano et al. (2000) [14]	Female, 55	Previously healthy	Sudden transient visual loss of the right eye, with subsequent diagnosis of cerebral infarction by MRI. Physical examination: unremarkable	Left ventricle, posterior wall, between the two papillary muscles; short stalk buried beneath the mass (5 mm long × 2 mm wide). Size: 2.5 cm in diameter	Two-dimensional and transesophageal echocardiography, magnetic resonance imaging approach: cardiopulmonary bypass and cardioplegia; transmitral route with a combined superior-transseptal incision; en bloc removal with a 5 mm margin of myocardium around the stalk	Typical histologic features of myxoma; no invasion in the resected myocardial cross-section	Outcome: uneventful. No evidence of recurrence. Follow-up: 24 months
Díaz and Aránguiz (2013) [9]	Female, 50	Arterial hypertension	Asymptomatic (incidental finding). Physical examination: grade III/VI systolic murmur at the aortic focus, auscultated during a routine hypertension check-up, which prompted the echocardiographic study	Mobile mass attached to the interventricular septum, causing partial left ventricular outflow tract (LVOT) obstruction. Size: 5.0 × 1.3 cm (2D echo); 2 cm anchoring stalk	Transthoracic echocardiography, cardiac magnetic resonance, and multidetector cardiac computed tomography. Approach: resection via transaortic transvalvular route, including the anchoring base, with a bovine pericardial patch	Calcified myxoma confirmed by histopathology	Outcome: not specified. Recurrence: not reported. Follow-up: not specified
Simsek et al. (2013) [15]	Male, 54	Chronic obstructive pulmonary disease. During hospitalization: diagnosis of pneumonia and congestive heart failure	Dyspnea, weakness, and fatigue attributable to heart failure. Physical examination: inspiratory crackles in the lower zones of both lung fields and pretibial edema	Pedunculated hanging mass on the interventricular septum, near the anterior mitral valve annulus; no gradient in the LVOT. Size: 3.3 × 1.2 cm (TEE); 3 × 1.3 cm (MRI)	Transthoracic and transesophageal echocardiography and magnetic resonance imaging; normal coronary angiography. Approach: transaortic route under cardiopulmonary bypass, with complete resection of the myxoma	Diagnosis of myxoma confirmed (gelatinous, fragile mass; no detailed microscopic description)	Outcome: postoperative left bundle branch block and atrial fibrillation; discharged on day 10. LVEF increased to 40%, and functional capacity improved from NYHA II-III to I. Recurrence: not reported. Follow-up: 12 months
Alvarado-Castro (2017) [2]	Male, 52	Dyslipidemia; chronic gastritis	Exertional dyspnea with retrosternal and epigastric pain of 2 months' duration. Physical examination: unremarkable	Left ventricle, posterior, and medial region of the apex; pedunculated tumor attached to the base of the infero-posterior papillary muscle. Size: 2.2 × 3.5 cm (TTE); 4 × 3 cm (intraoperative)	Transthoracic echocardiography, cardiac magnetic resonance imaging, and cardiac catheterization. Approach: aortic root opening and transvalvular route; resection and mechanical mitral valve replacement (Sorin #29) due to involvement of the mitral papillary base	Cardiac myxoma, no evidence of malignancy	Outcome: favorable, no complications; discharged on postoperative day 6. Recurrence: not reported. Follow-up: not specified
Orozco-Hernandez et al. (2019) [16]	Female, 55	Arterial hypertension, rheumatic fever, and coronary artery disease with recent intervention	Progressive dyspnea, orthopnea, palpitations, and functional decline. Physical examination: unremarkable	Left ventricle; mass attached to the septum and anterolateral wall, in continuity with the papillary muscles; intraoperatively, it occupied most of the LV cavity up to the apex, covered by thrombus, with calcifications, with a calcified anterior mitral leaflet and muscles fused to the mass. Size: not numerically specified	Transthoracic echocardiography, chest CT, cardiac magnetic resonance imaging, PET, and coronary angiography/ventriculography; confirmed by histology. Approach: balloon angioplasty of the LAD during angiography; right coronary artery revascularization with saphenous vein graft; superior transseptal approach; mitral valve resection and partial resection of the mass with a bioprosthetic valve; intra-aortic balloon pump for LV dysfunction	Histologic stains confirming myxoma with partially calcified thrombi	Outcome: timely extubation; balloon pump removed the next day; course complicated by arrhythmias (sinus bradycardia, transient AV block, atrial fibrillation, and cavotricuspid isthmus-dependent atrial flutter), with isthmus ablation. Recurrence: not reported. Follow-up: not specified
Mahavar et al. (2021) [17]	Female, 20	Surgical resection of left atrial myxoma in 2011 and 2015 (prior records unavailable). Family history negative in siblings or parents	Progressive dyspnea of 3 months (NYHA III), with an episode of syncope and numbness of both upper limbs. Physical examination: no relevant findings except a prior sternotomy scar	Attached to the ventricular aspect of the anterior mitral leaflet, swinging into the LVOT, stalk at the junction of the anterolateral papillary muscle and its chordae. Size: 20 × 19 mm (TTE); 21 × 18 mm (MRI)	Transthoracic echocardiography, cardiac magnetic resonance imaging, and intraoperative transesophageal echocardiography. Approach: aorto-bicaval cannulation, normothermic antegrade cardioplegia, and transverse aortotomy; en bloc removal without fragmentation or damage to the mitral apparatus	Histopathological examination confirming the diagnosis of myxoma	Outcome: uneventful. Recurrence: 6-month control echocardiogram with no recurrence. Follow-up: 6 months
Spiliopoulos et al. (2021) [18]	Male, 68	Malignant fibrous histiocytoma (right preauricular and left jugular regions) with regional relapse and right lung metastasis, both surgically treated; arterial hypertension, hypercholesterolemia, type II diabetes, smoking, and family history of coronary artery disease	ST-elevation myocardial infarction of the inferior wall; the day after coronary angiography, multiple cardiac arrests due to polymorphic ventricular tachycardia. Physical examination: unremarkable	Left ventricle; mass fixed to the septal wall, pedunculated, with a stalk easily shaved from the endocardium. Size: 6.2 × 4 cm (TTE); 6.7 cm (CT); 6.5 cm (intraoperative)	Transthoracic echocardiography, CT of chest/abdomen/pelvis (to exclude metastasis); scheduled TEE not performed due to clinical deterioration; confirmed by histology. Approach: urgent excision due to instability; median sternotomy, cardiopulmonary bypass, and antegrade cardioplegic arrest; left ventriculotomy 4 cm above the apex, parallel to the LAD; total excision of the pedunculated mass	Histologic examination confirming the diagnosis of myxoma	Outcome: uneventful; discharged on day 5. Recurrence: no signs of recurrence; normal echocardiograms during follow-up. Follow-up: 18 months
Li et al. (2022) [19]	Female, 68	Arterial hypertension	Exertional dyspnea. Physical examination: BP 140/80 mmHg; grade II-III systolic murmur at the mitral focus	Left ventricle, anterolateral wall. Size: approx. 1.5 × 1.2 cm (transesophageal echocardiography); 1.5 × 1.0 cm	Coronary CT angiography, cardiac magnetic resonance imaging, and transesophageal echocardiography. Approach: complete thoracoscopic resection; CPB via right femoral artery and vein; right atrial and interatrial septal incision; myxoma and stalk removed en bloc through the mitral valve	Myxoid areas and thrombus organization; final diagnosis of myxoma	Outcome: good recovery; discharged on postoperative day 7. Recurrence: not reported. Follow-up: 1 month (control echocardiogram with no residual mass or mitral valve abnormality)
Asad et al. (2023) [10]	Male, 25	Prior gastritis, treated with antibiotics for a positive *H. pylori *stool antigen	Chest pain of 3 months' duration. Physical examination: unremarkable	Left ventricle; attached by a 2-3 mm stalk to the mid-septum, 5 cm below the aortic annulus. Size: 2 × 2 cm encapsulated (echocardiography); 2 × 1.3 cm defect (CT angiography)	Echocardiography and chest CT angiography. Approach: median sternotomy, aorto-bicaval cardiopulmonary bypass, and normothermic antegrade blood cardioplegia; translucent, fragile, jelly-like tumor visualized through the aortic cusps, attached to the septum by a stalk, transected with a harmonic scalpel, and removed en bloc	Histopathological examination consistent with cardiac myxoma	Outcome: uneventful; discharged on postoperative day 4; complete resolution of symptoms. Recurrence: no recurrence during follow-up. Follow-up: 6 months
Liu et al. (2025) [20]	Male, 49	No medical history	Asymptomatic (incidental finding on routine transthoracic echocardiography). Physical examination: unremarkable	Left ventricle, midlateral wall, adjacent to the anterior papillary muscle, with ill-defined borders; intraoperatively adherent to the papillary muscle and chordae tendineae, with invasive growth into the wall. Size: 27.5 × 16.5 mm (TTE); 33 × 19 × 20 mm (MRI); 35 × 30 × 30 mm (intraoperative)	Multimodal imaging (TTE and contrast echocardiography, CT, cardiac magnetic resonance imaging, and 18F-FDG PET/CT); confirmed by histopathology and immunohistochemistry. Approach: subtotal resection due to extensive adhesion to the myocardium, papillary muscle, and chordae, to preserve the mitral valve	LV myxoma with pseudoinvasive entrapment of the papillary muscle, without destructive invasion. Immunohistochemistry: SMA+, CD34+++, CD31+++; Ki-67 1%	Outcome: satisfactory recovery; discharged after one week. Recurrence: no evidence of recurrence. Follow-up: 23 months
Yamazaki et al. (2025) [21]	Female, 39	No medical history	Sudden-onset abdominal and right leg pain (acute arterial occlusion). Physical examination: cold, cyanotic right lower extremity, with a difficult-to-palpate femoral artery	Pedunculated tumor inserted on the interventricular septum. Size: 8 × 30 mm (TTE); 3 × 3 cm giant jelly-like (intraoperative)	Transthoracic echocardiography confirmed by histopathology. Approach: median sternotomy with cardiopulmonary bypass and antegrade cardioplegia; ascending aortotomy, incision between the LAD and the diagonal artery to access the LV; tumor resected en bloc from its insertion; cryoablation of the margin and LV wall repair with Teflon felt strips	Typical histopathological findings of cardiac myxoma	Outcome: complicated by myonephropathic metabolic syndrome with compartment syndrome of the right lower limb (required continuous hemodiafiltration, discontinued on day 5); postoperative CT with good flow. Recurrence: no recurrence. Follow-up: 3 years
Wu et al. (2026) [22]	Female, 53	Cervical cancer treated with 28 sessions of radiotherapy and three cycles of chemotherapy	Acute right-sided hemiparesis and dysarthria of 2 h (first event); readmission at 2 weeks for syncope and dyspnea. Physical examination: BP 150/105 mmHg, tachycardia 104 bpm, RR 30/min, decreased heart sounds, no murmurs	Left ventricle; mobile polypoid mass attached to the lateral wall by a 2-3 mm stalk. Size: 20.3 × 13.4 mm (echocardiography)	Transthoracic echocardiography. Approach: not performed; surgical excision was scheduled, but the patient died before the intervention	Not performed. Diagnosis of LV myxoma was established without pathological confirmation, based on the characteristic echocardiographic findings	Outcome: fatal outcome on hospital day 7 (no surgery performed). Recurrence: not applicable. Follow-up: not applicable (in-hospital preoperative death)

## Conclusions

Cardiac myxomas are rare, usually benign, primary tumors, and their presentation in the left ventricle is exceptional. This case illustrates an atypical presentation in a young patient with nonspecific symptoms, highlighting the importance of maintaining a high index of clinical suspicion for intracardiac masses, even in nonsuggestive clinical contexts.

Echocardiography remains the fundamental initial diagnostic tool, complemented by other imaging modalities that allow for adequate characterization and therapeutic planning. Timely surgical resection remains the treatment of choice, with excellent results when performed early. Overall, this case emphasizes the relevance of a comprehensive and timely diagnostic approach for unusual presentations of cardiac myxoma, with the aim of preventing potentially serious complications and optimizing the patient's prognosis.
